# Meta-Analysis of Exploring the Effect of Curcumin Supplementation with or without Other Advice on Biochemical and Anthropometric Parameters in Patients with Metabolic-Associated Fatty Liver Disease (MAFLD)

**DOI:** 10.3390/ijerph20054266

**Published:** 2023-02-27

**Authors:** Gracjan Różański, Hanna Tabisz, Marta Zalewska, Wojciech Niemiro, Sławomir Kujawski, Julia Newton, Paweł Zalewski, Joanna Słomko

**Affiliations:** 1 Department of Exercise Physiology and Functional Anatomy, Ludwik Rydygier Collegium Medicum in Bydgoszcz Nicolaus Copernicus University in Torun, Świętojańska 20, 85-077 Bydgoszcz, Poland; 2 Department of Prevention of Environmental Hazards and Allergology, Medical University of Warsaw, 02-091 Warsaw, Poland; 3 Faculty of Mathematics, Informatics and Mechanics University of Warsaw, 02-097 Warsaw, Poland; 4 Faculty of Mathematics and Computer Science, Nicolaus Copernicus University, 87-100 Torun, Poland; 5 Population Health Sciences Institute, The Medical School, Newcastle University, Framlington Place, Newcastle-upon-Tyne NE2 4HH, UK; 6 Laboratory of Centre for Preclinical Research, Department of Experimental and Clinical Physiology, Warsaw Medical University, 1b Banacha Street, 02-097 Warsaw, Poland

**Keywords:** alternative medicine, MAFLD, curcumin, supplementation, liver

## Abstract

Metabolic (dysfunction)-associated fatty liver disease (MAFLD), previously known as non-alcoholic fatty liver disease (NAFLD), is the most common chronic liver disease. MAFLD is characterized by the excessive presence of lipids in liver cells and metabolic diseases/dysfunctions, e.g., obesity, diabetes, pre-diabetes, or hypertension. Due to the current lack of effective drug therapy, the potential for non-pharmacological treatments such as diet, supplementation, physical activity, or lifestyle changes is being explored. For the mentioned reason, we reviewed databases to identify studies that used curcumin supplementation or curcumin supplementation together with the use of the aforementioned non-pharmacological therapies. Fourteen papers were included in this meta-analysis. The results indicate that the use of curcumin supplementation or curcumin supplementation together with changes in diet, lifestyle, and/or physical activity led to statistically significant positive changes in alanine aminotransferase (ALT), aspartate aminotransferase (AST), fasting blood insulin (FBI), homeostasis model assessment of insulin resistance (HOMA-IR), total triglycerides (TG), total cholesterol (TC), and waist circumference (WC). It appears that these therapeutic approaches may be effective in alleviating MAFLD, but more thorough, better designed studies are needed to confirm this.

## 1. Introduction

**Figure 1:**
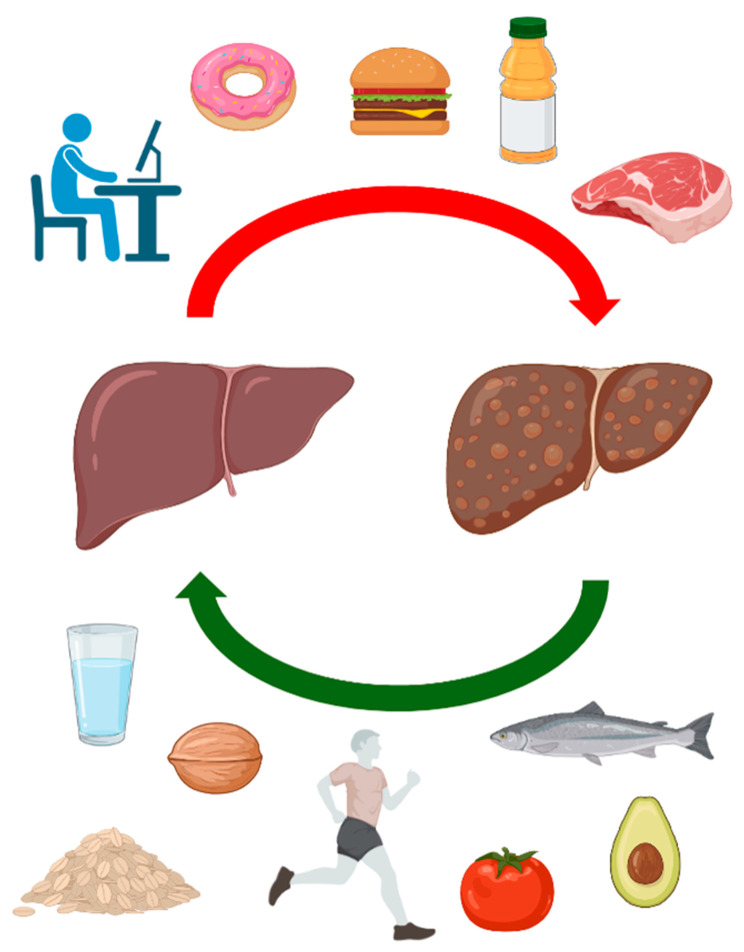
Modifiable factors negatively affecting liver health (upper part of the figure)—sweetened drinks, saturated fatty acids, sweets, processed foods, and a sedentary lifestyle, and modifiable factors positively affecting liver health (lower part of the figure)—vegetables, fruit (low in fructose), mono- and polyunsaturated fatty acids, fiber, and physical activity. Created with BioRender.com.

Metabolic-associated fatty liver disease (MAFLD), formerly known as non-alcoholic fatty liver disease (NAFLD) is the most common chronic liver disease worldwide [1,2]. It is becoming a huge public health problem as its prevalence is on the rise, generating large costs (the estimated annual cost in Europe is EUR 35 billion, while it is EUR 89 billion in the US) [3,4]. MAFLD is characterized by excessive (>5% of liver weight) fat accumulation in hepatocytes that is not caused by a viral infection, alcohol consumption, or medication [5]. In addition, the condition coexists with other diseases or metabolic disorders such as being overweight or obese, type 2 diabetes or pre-diabetes, insulin resistance, dyslipidemia, or hypertension [6,7,8,9] and is also a factor that increases the risk of liver- and cardiovascular-disease-related mortality [10,11]. Another serious risk is the possibility of disease progression to non-alcoholic steatohepatitis (NASH), which may occur in 23–44% of MAFLD patients, resulting in fibrosis and even cirrhosis, which within 5–7 years leads to liver failure in 40–60% of cases and within 3–7 years to hepatocellular carcinoma (HCC) in 2.4–12% of patients [12]. In view of the potential progression of MAFLD and the high costs, early diagnosis, prevention, treatment of risk factors, and lifestyle modification are important (Figure 1) [3]. In 2016, the European Association for the Study of the Liver specifically recommended dietary changes and a gradual increase in aerobic exercise or resistance training as interventions leading to lifestyle changes in patients with MAFLD. The recommendations for diet and physical activity, among other reasons, are due to the fact that no effective pharmacological therapy is currently available [13].

The aim of this study is to review the effects of curcumin supplementation only or combined with dietary, physical activity, and/or lifestyle changes on biochemical and anthropometric parameters in the course of MAFLD. The indicated aim of the study is based on the existing knowledge of the pathomechanism of MAFLD and the known therapeutic properties of curcumin, whose (disease pathomechanism and curcumin properties) are described in the following section.

## 2. Pathophysiology

It is now recognized that factors leading to MAFLD include a poor diet, a sedentary lifestyle, and genetic and environmental factors. Therefore, the mechanisms that lead to the development of MAFLD are complex, leading to it being referred to as the “multiple hits hypothesis”. Although current knowledge points to specific factors leading to MAFLD, the exact pathomechanism is not yet fully understood. However, its basis is considered to be insulin resistance (IR), which results in increased de novo hepatic lipogenesis (DNL) and reduced inhibition of adipose tissue lipolysis, leading to the increased influx of fatty acids (FA) into hepatocytes and their storage as triglycerides. In addition, IR also causes dysfunction of adipose tissue resulting in altered production and secretion of adipokines and proinflammatory cytokines. High levels of free fatty acids, free cholesterol, and other lipid metabolites result in lipotoxicity, which is also an important part of the pathomechanism of MAFLD. This leads to increased levels of reactive oxygen species, which cause dysfunction of the endoplasmic reticulum and mitochondria. Changes in the intestinal microbiota may also be involved in the increased levels of free fatty acids, leading to increased permeability of the small intestine, which results in enhanced absorption of FA. This results in the activation of pro-inflammatory pathways and the release of pro-inflammatory cytokines such as IL-6 and TNF-α [6].

## 3. Curcumin

Curcumin is a polyphenol belonging to the group of curcuminoids. It occurs in the rhizomes of the plant called turmeric (*Curcuma longa*), which belongs to the ginger family. Turmeric is naturally found in Asia, mainly in India. It is mainly known for its culinary applications due to its taste, aroma, and intense yellow color. However, turmeric has been used in medicine for thousands of years due to its curcumin content [14]. Curcumin is characterized by many desirable properties. It has anti-inflammatory, antioxidant, and anticancer properties, among others [15]. Furthermore, importantly, it is safe and rarely causes adverse symptoms. For this reason, it is used to treat or support the treatment of many diseases, e.g., cardiovascular diseases, inflammatory bowel diseases, breast, stomach, pancreatic and lung tumors, dermatoses, allergic asthma, and liver diseases [16,17,18,19,20,21].

## 4. Material and Methods

The protocol for this systematic review and meta-analysis was based on the preferred reporting items of systematic reviews and meta-analysis (PRISMA) statement [22]. The design of the present work was fully specified in advance. It was registered in the PROSPERO (International Prospective Register of Systematic Reviews, CRD42022310950, https://www.crd.york.ac.uk/prospero/display_record.php?RecordID=310950, accessed on 24 February 2023).

### 4.1. Types of Participants

Participants meeting the inclusion criterion of being adults (>18 years old) suffering from MAFLD were eligible for the study group. No additional criteria, such as gender or nationality, were defined. Participants were excluded from the study when they did not manifest MAFLD.

### 4.2. Types of Interventions

Interventions using curcumin supplementation only or curcumin supplementation with other changes e.g., diet and/or physical activity and/or other lifestyle modifications) and presenting the results of biochemical and/or anthropometric parameters’ outcomes before and after supplementation were included. Studies involving animals were excluded.

### 4.3. Types of Comparisons

There were no specific comparison criteria.

### 4.4. Types of Outcomes

The outcome of at least one biochemical parameter or anthropometric measurement is presented in the study, measured at baseline (pre-intervention) and post-intervention. The biochemical and anthropometric parameters measured in each study are presented in Table 1 and Table 2.

### 4.5. Types of Studies

Only randomized controlled trials published in peer-reviewed journals in English were included. The precise duration of the undertaken intervention was not specified. The exclusion criterion was a non-human study. The detailed PICOS criteria are described in Table 3.

### 4.6. Search Strategy and Study Selection

**Figure 2:**
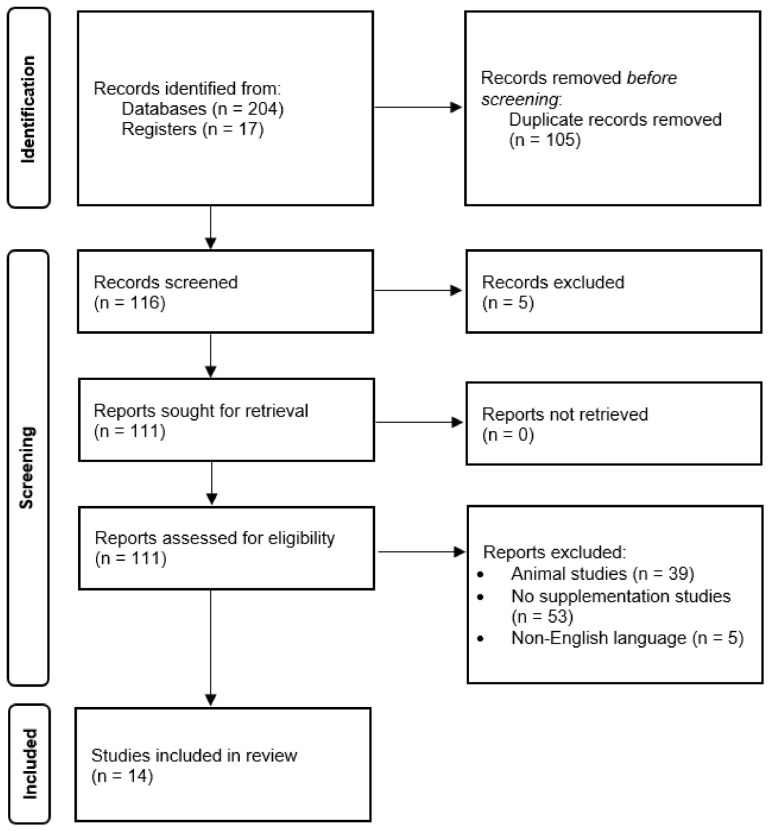
PRISMA flow diagram of the study selection [22].

We reviewed available publications using databases such as PubMed, Web of Science, and Scopus using the words “NAFLD” or “MAFLD” “metabolic-associated fatty liver disease” or “non-alcoholic fatty liver disease” and “curcumin” or “turmeric”. We limited the results to papers in English and published by March 2022 (Figure 2).

### 4.7. Quality and Risk of Bias Assessment

An assessment of the quality of the studies meeting the inclusion criteria was performed using the Cochrane risk of bias tools. The following elements of the studies were analyzed: selection bias (random sequence generation and allocation concealment), performance bias (blinding of participants and personnel), detection bias (blinding of outcome assessment), attrition bias (incomplete outcome data), reporting bias (selective reporting), and other bias (Table 4) [37]. Funnel plots have been used to provide a visual assessment of the association between treatment estimate and study size. Publication bias was considered significant when the *p*-value was less than 0.05 in either Begg’s test [38] (Supplementary Figures S1 and S2).

### 4.8. Statistical Analysis

The meta-packages of R were used to perform the analyses [38,39,40,41]. A random effects model was conducted to estimate the pooled effect, for values of I2 ≥ 50%, while a fixed effects model was conducted for values of I2 ≤ 50%. The effect size was calculated as the mean difference (MD) changes from baseline along with 95% confidence intervals (CI). A *p*-value < 0.05 was defined as statistically significant. The results of the conducted analyses are presented as a forest plot. The heterogeneity among the included studies was evaluated by the I2 statistic. An I2 value of >50% corresponds to high heterogeneity, values between 25–50% define heterogeneity as moderate, while I2 < 25% indicates low heterogeneity.

Cytoscape (version: 3.8.1) was used to create network graphs presenting the studies’ results [42].

**Table 4:** Quality of the trials and Cochrane risk bias [43].

	Random Sequence Generation	Allocation Concealment	Blinding of Participant and Personnel	Blinding of Outcome Assessment	Incomplete Outcome Data	Selective Reporting	Other Bias
Rahmani, 2016 [23]	+	+	+	+	+	+	+
Kelardeh, 2017 [24]	+/−	+	+	+	+	+	+/−
Navekar, 2017 [25]	+	+	+	+	+	+	+
Chashmniam, 2019 [26]	+	+	+	+	+	+	+
Mirhafez, 2019 [27]	+/−	+	+	+	+	+	+
Hariri, 2020 [28]	+	+	+	+	+	+	+
Kelardeh, 2020 [29]	+/−	+	+	+	+	+	+/−
Saberi-Karimian, 2020 [30]	+	+	+	+	+	+	+
Panahi, 2016 [31]	+/−	+	−	−	+	+	+
Panahi, 2017 [32]	+	+	+	+	+	+	+
Jazayeri-Tehrani, 2019 [33]	+/−	+	+	+	+	+	+
Saadati, 2019 (a) [34]	+	+	+	+	+	+	+
Saadati, 2019 (b) [35]	+	+	+	+	+	+	+
Cicero, 2020 [36]	+	+	+	+	+	+	+

+ Low risk of bias. +/− Unclear risk of bias. − High risk of bias.

## 5. Results

### 5.1. Study Selection

In total, 221 studies were analyzed to meet the inclusion criteria. Ultimately, 14 randomized controlled trials were included in this meta-analysis. Table 1 and Table 2 show the characteristics of the trials included in the meta-analysis including a division by type of applied intervention.

### 5.2. Participant and Study Characteristics

Eight hundred and seventy-four NAFLD patients (429 in the treatment group and 418 in the control group) were included in the meta-analysis. Table 1 shows the characteristics of the studies in which only curcumin was supplemented. Table 2 presents the characteristics of studies in which, in addition to curcumin supplementation, physical activity and/or dietary advice and/or lifestyle changes were also used.

### 5.3. Interventions

In 8 of the study groups included in the analysis, only curcumin supplementation was used; in the other 8 studies groups, curcumin supplementation was also used, but combined with physical activity and/or dietary advice and/or lifestyle changes. Two studies out of fourteen used both of the abovementioned types of intervention; therefore, the total number of study groups amounted to 16. The duration of the intervention was 8 or 12 weeks. The doses of curcumin used ranged from 80–1500 mg/day. Detailed characteristics of the studies are presented in Table 1 and Table 2.

### 5.4. Effect of Curcumin Supplementation and Curcumin Supplementation with Physical Activity and/or Dietary Advice and/or Lifestyle Changes on the Levels of Biochemical Parameters

ALT and AST levels were controlled in nine studies [23,24,26,27,28,32,33,34,35]. Fasting blood insulin levels (FBI) and HOMA-IR were controlled in five studies [25,31,33,35,36]. TG, TC, and LDL-C levels were controlled in eight studies [23,26,27,30,31,33,35,36]. Waist circumference (WC) was controlled in seven studies [24,28,30,32,33,35,36].

**Figure 3:**
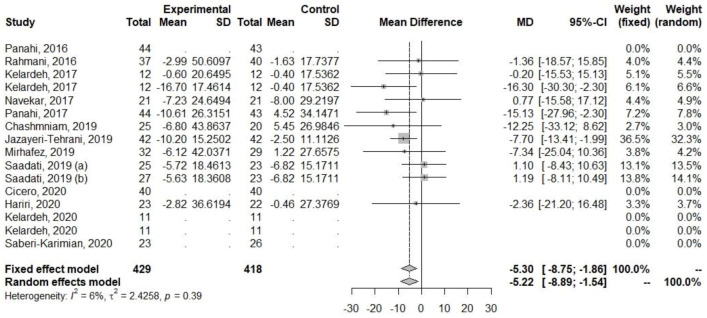
Effect of curcumin supplementation and curcumin supplementation with other advice (**left**) vs. control (**right**) on ALT levels [23,24,25,26,27,28,32,33,34,35].

**Figure 4:**
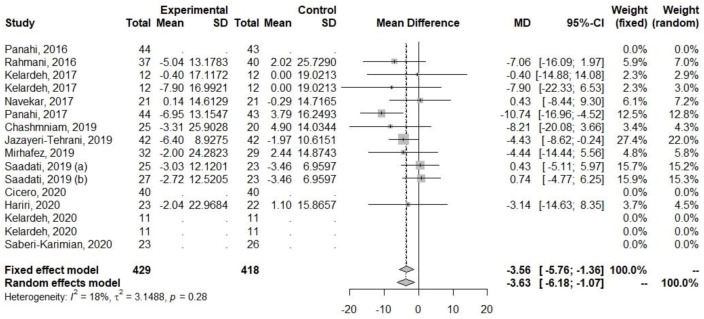
Effect of curcumin supplementation and curcumin supplementation with other advice (**left**) vs. control (**right**) on AST levels [23,24,25,26,27,28,32,33,34,35].

Regarding ALT and AST, decreases in their levels were observed (Table 5, Figure 3 and Figure 4). The heterogeneity of the effect measures regarding ALT (I2 = 6.0%, *p* = 0.39) and AST (I2 = 17.5%, *p* = 0.28) was low.

**Figure 5:**
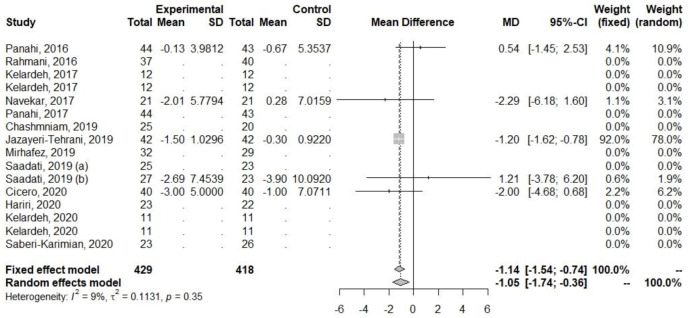
Effect of curcumin supplementation and curcumin supplementation with other advice (**left**) vs. control (**right**) on FBI levels [25,31,33,35,36].

**Figure 6:**
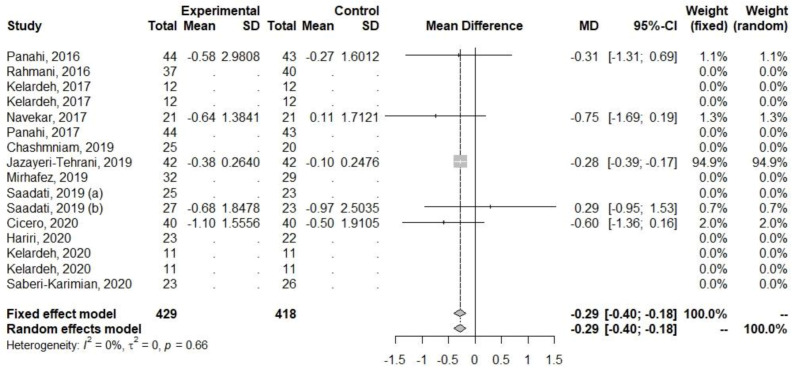
Effect of curcumin supplementation and curcumin supplementation with other advice (**left**) vs. control (**right**) on HOMA-IR [25,31,33,35,36].

Decreases were also observed for parameters related to glucose metabolism (FBI and HOMA-IR), Table 5, Figure 5 and Figure 6. The heterogeneity for FBI (I2 = 9.3%, *p* = 0.35) and HOMA-IR (I2 = 0%, *p* = 0.66) was low.

**Figure 7:**
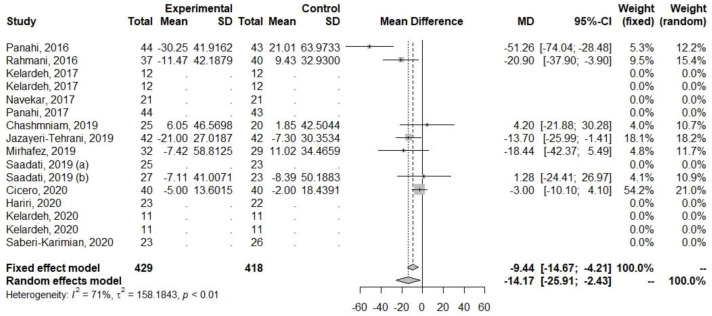
Effect of curcumin supplementation and curcumin supplementation with other advice (**left**) vs. control (**right**) on LDL-C levels [23,26,27,31,33,35,36].

**Figure 8:**
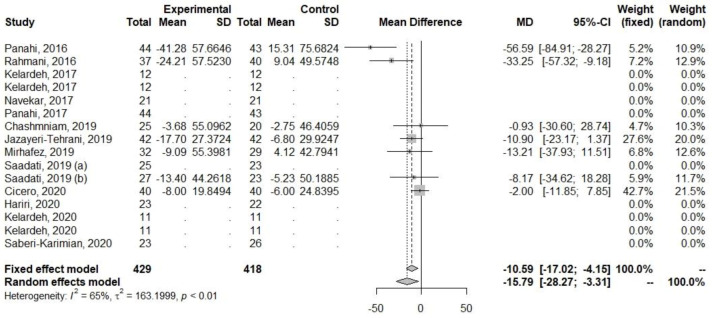
Effect of curcumin supplementation and curcumin supplementation with other advice (**left**) vs. control (**right**) on total TC levels [23,26,27,31,33,35,36].

**Figure 9:**
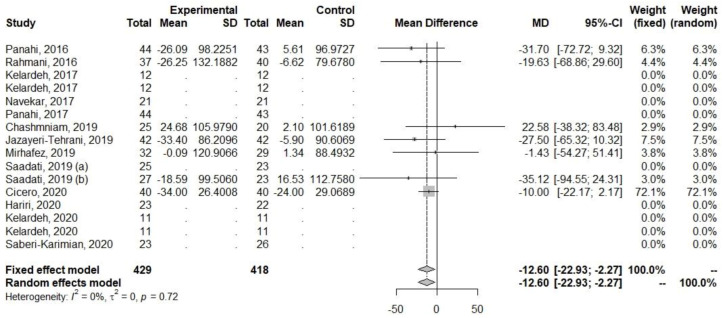
Effect of curcumin supplementation and curcumin supplementation with other advice on (**left**) vs. control (**right**) TG levels [23,26,27,31,33,35,36].

Decreases in levels were also observed among parameters related to lipid metabolism (TG, TC, and LDL-C), Table 5, Figure 7, Figure 8 and Figure 9. The heterogeneity for TG (I2 = 0%, *p* = 0.72) was low, but for TC (I2 = 64.6%, *p* < 0.01) and LDL-C (I2 = 70.8%, *p* < 0.01), it was high.

**Figure 10:**
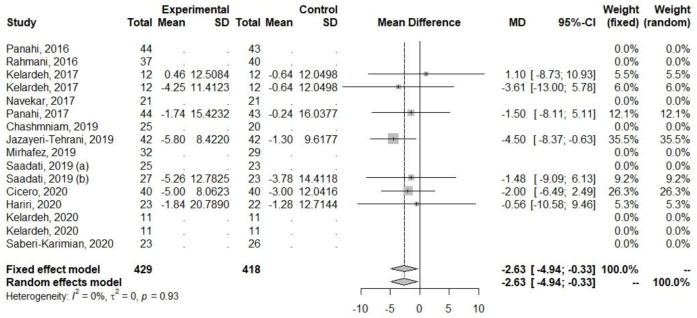
Effect of curcumin supplementation and curcumin supplementation with other advice on (**left**) vs. control (**right**) WC [24,28,32,33,35,36].

Among the anthropometric parameters, a reduction in WC was observed (Table 5, Figure 10). The heterogeneity for WC (I2 = 0%, *p* = 0.93) was low.

**Figure 11:**
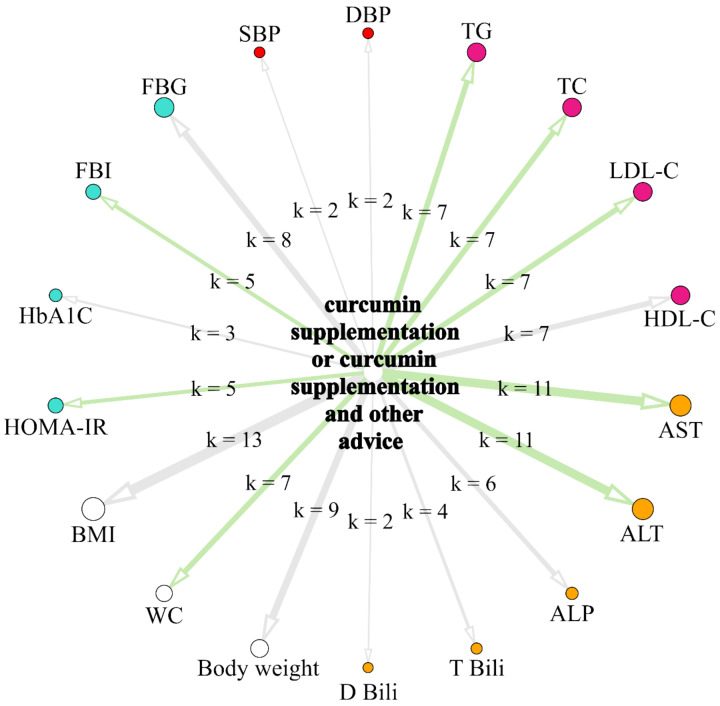
Network graph summarizing the results from random effects models assessing separately the effects of supplementation with curcumin with or without other advice on biochemical and anthropometric parameters.

The network presented in Figure 11. summarizes the results from single random effects models. The size of circular nodes is proportionally related to the overall sample size of the intervention groups from studies included in the model assessing the effects of the intervention on the parameter. The color of the node denotes the parameters group (white for anthropometrical parameters, orange for liver-function-related parameters, ruby for c, red for blood pressure indicators, and turquoise for parameters related to glucose and insulin metabolism). The width of the arrows is proportional to the number of studies included into a model assessing the effects of the intervention on the particular parameter (k), which is also denoted as a label. The color of the arrows indicates the results of random effects models: light green arrows denote statistically significant beneficial effects of supplementation with curcumin with or without other advice on parameters, while light grey arrows denote statistically non-significant effects.

## 6. Discussion

Our meta-analysis summarizes the findings of fourteen RCTs that used curcumin supplementation or curcumin supplementation with physical activity and/or dietary advice and/or lifestyle changes. The studies conducted to date using curcumin indicate its many positive effects in the course of numerous diseases [14,44]. Physical activity, part of a broader lifestyle, also has many health benefits [45]. Recommendations from the World Health Organization (WHO) indicate that adults, in order to maintain optimal health, should perform at least 150–300 min of moderately intense exercise or 75–150 min of vigorous exercise weekly [46]. Diet is also an important component affecting health, and in the context of MAFLD, poor diet is one of the key elements leading to the development of the disease [6,47]. Due to the lack of an effective drug therapy for MAFLD, attempts are being made to use supplementation, diet, physical activity, and lifestyle changes as treatment, but it is not clear which combinations of the aforementioned elements of therapy would give the greatest effectiveness.

Our results indicate that in many studies, in addition to curcumin supplementation, patients with MAFLD were also advised on changing their diet and lifestyle or implementing physical activity. This is a very important fact in the context of interpreting the results of the studies, as each of these elements may additionally influence the change in the parameters studied making it impossible to unequivocally assess the efficacy of curcumin supplementation. Therefore, our meta-analysis highlighted the fact that, in the indicated studies, other types of interventions were used in addition to curcumin supplementation.

Our results suggest that curcumin supplementation or curcumin supplementation together with a combined change in dietary habits and/or implementation of physical activity and/or lifestyle changes causes a decrease in ALT, AST, FBI, LDL-C, TC, and TG and a decrease in HOMA-IR and WC levels. 

The results of our study are in keeping with previous meta-analyses in several cases, but there are also a few differences in the results. In the case of ALT, our results are consistent with the studies of Ngu et al. [48], Goodarzi et al. [49], Yang et al. [50], and Jalali et al. [51], who also reported statistically significant decreases. In contrast, Wei et al. [52] obtained a decrease that was not statistically significant, but it should be noted that only two studies were included in the analysis. In the case of AST, our results are in accordance with all of the aforementioned publications [48,49,50,51,52], in which the authors also reported statistically significant decreases. FBI has so far been analyzed in two meta-analyses. Jalali et al. [51] reported a statistically significant decrease, which is consistent with our results, while Wei et al. [52] reported a decrease, but it was not statistically significant. For HOMA-IR, we reported a statistically significant decrease, similar to Yang et al. [50], Jalali et al. [51], and Wei et al. [52] in their meta-analyses. Among the lipid metabolism parameters (TG, TC, and LDL-C), decreases have been reported in previous meta-analyses, but they have not always been statistically significant. For TG, Yang et al. [50], Jalali et al. [51], and Wei et al. [52] also obtained statistically significant decreases in their analyses, while Ngu et al. [48] reported a statistically insignificant decrease. Regarding TC, Ngu et al. [48], Yang et al. [50], and Jalali et al. [51] obtained statistically significant decreases, which was also reported in our study. In contrast, Wei et al. [52] reported a statistically insignificant decrease in TC. For LDL-C, we obtained a statistically significant decrease, as well as Jalali et al. [51] and Wei et al. [52], while Ngu et al. [48] and Yang et al. [50] reported statistically insignificant decreases. Waist circumference has only previously been analyzed in the study of Baziar et al. [53], who obtained a statistically significant decrease.

Our study has several strengths. First, it provides evidence of the positive effects of curcumin supplementation and curcumin supplementation with added physical activity and/or dietary recommendations and/or lifestyle changes on the levels of certain blood biochemical parameters and waist circumference.

Our study includes more RCTs than most previously published meta-analyses and also highlights the fact that some studies used other interventions (dietary recommendations, physical activity, and lifestyle changes) in addition to curcumin supplementation, which, to the best of our knowledge, has been omitted in previous publications.

In opposition to the strengths, there are some limitations. Firstly, not all of the studies controlled for the same biochemical parameters. Secondly, there were differences in the duration of the different interventions. Third, the doses of curcumin used and the form of curcumin varied between studies. Fourth, additional recommendations for curcumin supplementation were not always described in detail, with the information being limited to only general information about their type. Fifth, the study groups, especially in some studies, were small.

## 7. Conclusions

This meta-analysis, based on RCTs, provides evidence that curcumin supplementation only or curcumin supplementation with physical activity and/or dietary advice and/or lifestyle changes lead to decreases in ALT, AST, FBI, LDL-C, TC, and TG in blood levels and a decrease in HOMA-IR and WC levels. However, this was due to the use of curcumin doses in the range of 80–1500 mg and additional recommendations, which were not always described in detail. The studies conducted to date do not clearly identify the appropriate dose of curcumin, either used alone or in combination with additional physical activity and/or diet and/or lifestyle recommendations. It is also not possible to determine, on the basis of current studies, the effect of the mentioned additional recommendations on the effect induced by curcumin and therefore also its dose.

Therefore, further well designed studies among MAFLD patients, using curcumin only and with additional recommendations, are needed. The effect of physical activity, diet and lifestyle on the effect induced by curcumin supplementation is also worthy of analysis. An element to be taken into consideration in future studies is the dose of curcumin. Other factors that may influence the results of the study, such as the diet, physical activity, lifestyle, or education of the patients participating in the study, should be considered and described in detail. It is important that the aforementioned interventions are detailed and communicated to the participants to ensure that the patients follow the recommendations with full understanding and according to the established rules, as this may affect the final results. Recommendations cannot be based on general indications, such as “increase physical activity” or “follow a healthy diet”, as this is a strong limitation of the study, with it not allowing for an accurate assessment of the impact of the applied interventions. Each recommendation should be precisely defined, preferably (where possible) in a measurable way, such as ‘30 min a day of walking 5 times a week’ or ‘consume 200 g of salmon per week’. Through the subjects following specific guidelines, it prevents variation in the interventions used, within the group, due to misunderstanding or patients’ own interpretation of the recommendations. Measurable recommendations also make it possible to assess the extent to which patients have followed them.

In conclusion, despite the limitations of the studies carried out to date, it seems that only curcumin supplementation or with the addition of physical activity and/or dietary advice and/or lifestyle changes can be helpful in the treatment of patients with MAFLD.

## Figures and Tables

**Table 1:** Research using only supplementation.

	Study Design	Duration	n (Study Group/ Control Group)	Age, Years	BMI	Dose	Control Group	**Tested Parameters**
Rahmani, 2016 [23]	RCT	8 weeks	37/40	46.3 ± 11.57	30.84 ± 4.45	500 mg/day of an amorphous dispersion preparation comprising 70 mg curcuminoids	Received placebo	ALT, AST, BMI, FGB, HbA1c, HDL-C, LDL-C, TC, TG, body weight
Kelardeh, 2017 [24]	RCT	12 weeks	12/12	37.41 ± 5.17	29.88 ± 4.49	80 mg/day curcumin as a nanomicelle	Received one placebo (3 g dextrose) capsule per day	ALT, AST, ALP, BMI, WC
Navekar, 2017 [25]	DB, RCT	12 weeks	21/21	42.09±7.23	31.81 ± 4.58	3 g/day turmeric (6 × 500 mg three times a day)	Patients were given six placebo capsules daily	ALT, AST, BMI, FGB, FBI, HOMA-IR, body weight
Chashmniam, 2019 [26]	DB, RCT	8 weeks	25/20	46.56 ± 2.25	30.03 ± 0.7	Phospholipid curcumin 250 mg/day (equivalent to 50 mg pure curcumin)	Received one placebo capsule per day	ALT, AST, ALP, BMI, D Bili, FGB, HDL-C, LDL-C, TC, TG, T Bili, body weight
Mirhafez, 2019 [27]	DB, RCT	8 weeks	32/29	44.8 ± 11.14	30.06 ± 5.76	Phospholipid curcumin 250 mg/day (equivalent to 50 mg pure curcumin)	Received a placebo	ALT, AST, BMI, FGB, HDL-C, LDL-C, TC, TG, body weight
Hariri, 2020 [28]	DB, RCT	8 weeks	23/22	40.95 ± 12.24	30.59 ± 5.91	Phospholipid curcumin 250 mg/day (equivalent to 50 mg pure curcumin)	Received a placebo	ALT, AST, BMI, WC, body weight
Kelardeh, 2020 [29]	RCT	12 weeks	11/11	66.72 ± 3.03	27.60 ± 1.26	80 mg/day curcumin as a nanomicelle	Received a placebo capsule	ALP, BMI, T Bili, body weight
Saberi-Karimian, 2020 [30]	RCT	8 weeks	23/26	18–70	30.02 ± 5.45	500 mg curcuminoids + 5 mg piperine/day	Received one placebo capsule per day	ALT, AST, TG, TC, LDL-C, HDL-C, BDP, SBP, FBG, WC, BMI, body weight

ALP, alkaline phosphatase; ALT, alanine aminotransferase; AST, aspartate aminotransferase; BMI, body mass index; Creat, creatinine; CT, clinical trial; DB, double blind; DBP, diastolic blood pressure; D Bili, direct bilirubin; FGB, fasting blood glucose; FBI, fasting blood insulin; HbA1c, glycated hemoglobin; HDL-C, high-density lipoprotein cholesterol; HOMA-IR, homeostasis model assessment of insulin resistance; LDL-C, low-density lipoprotein cholesterol; RCT, randomized controlled trial; SBP, systolic blood pressure; TC, total cholesterol; TG, total triglycerides; T Bili, total bilirubin; WC, waist circumference.

**Table 2:** Studies using supplementation and other advice.

	Study Design	Duration	n (Study Group/ Control Group)	Age, Years	BMI	Dose	Other Advice	Control Group	Tested Parameters
Panahi, 2016 [31]	RCT	8 weeks	44/43	45 ± 12.59	28.97 ± 3.42	1000 mg/day in two divided doses	All patients received dietary and lifestyle advice before the start of the trial	Patients received lactose as a placebo	FGB, FBI, HbA1c, HDL, HOMA-IR, LDL-C, TC, TG
Kelardeh, 2017 [24]	RCT	12 weeks	12/12	38.24 ± 6.59	30.27 ± 4.34	80 mg curcumin as a nanomicelle	12 weeks of non-linear resistance training. Each session took about 45–60 min, three days a week (non-consecutive), lasting 12 weeks	Received one placebo capsule per day	ALP, ALT, AST, BMI, WC
Panahi, 2017 [32]	RCT	8 weeks	44/43	44.98 ± 12.59	28.97 ± 3.42	1000 mg/day in 2 divided doses	All patients received dietary and lifestyle advice before the start of the trial	Received a placebo	ALP, ALT, AST, BMI, D Bili, T Bili, WC
Jazayeri-Tehrani, 2019 [33]	DB, RCT	12 weeks	42/42	41.8 ± 5.6	30.6 ± 2.14	The sinacurcumin^®^ dose was 80 mg/day (two 40 mg capsules per day)	The lifestyle advice was equally presented by a trained dietician (SAJT) at the hospital	Received two placebo capsules per day	ALT, AST, BMI, DBP, FGB, FBI, HbA1c, HDL-C, HOMA-IR, LDL-C, SBP, TC, TG, WC, Body weight
Saadati, 2019 (a) [34]	RCT	12 weeks	25/23	45.13 ± 10.9	32.30 ± 4.55	3 × 500 mg/day (95% curcuminoids)	Nutrient distribution was as follows: diet macronutrients 52 to 55% of energy from carbohydrates, less than 30% of energy from lipids, and 15–18% of energy from proteins was provided. Patients were advised to exercise ≥30 min, three times per week.	Received three placebo capsules per day	ALT, AST
Saadati, 2019 (b) [35]	RCT	12 weeks	27/23	45.13 ± 10.9	32.3 ± 4.55	3 × 500 mg/day (95% curcuminoids)	The distribution of nutrients: total fat less than 30% total energy value, protein 15–18%, and carbohydrates 52–55%. Patients are advised to exercise ≥30 min, three times per week	Received placebo capsules	ALT, AST, BMI, FGB, FBI, HDL-C, HOMA-IR, LDL-C, TC, TG, WC, Body weight
Cicero, 2020 [36]	DB, RCT	8 weeks	40/40	54 ± 3	27.1 ± 1.8	800 mg phytosomal curcumin (Curserin^®^: 200 mg curcumin, 120 mg phosphatidylserine, 480 mg phosphatidylcholine, and 8 mg piperine from *Piper nigrum* L. dry extract)	The patients were advised to follow the recommendations of the Mediterranean diet. Additionally, they were advised to engage in physical activity by walking briskly for 20–30 min, 3–5 times per week, or by cycling	Patients received a placebo	BMI, DBP, FGB, FBI, HDL-C, HOMA-IR, LDL-C, SBP, TC, TG, WC
Kelardeh, 2020 [29]	RCT	12 weeks	11/11	64.09 ± 3.33	27.03 ± 0.65	80 mg curcumin as a nanomicelle	The nonlinear resistance training program: each session took about 60–70 min for the main training (plus about 20 min for the warm-up and cool-down), three days a week (non-consecutive) which lasted 12 weeks.	Received one placebo capsule per day	ALP, BMI, T Bili, Body weight

ALP, alkaline phosphatase; ALT, alanine aminotransferase; AST, aspartate aminotransferase; BMI, body mass index; Creat, creatinine; CT, clinical trial; DB, double blind; DBP, diastolic blood pressure; D Bili, direct bilirubin; FGB, fasting blood glucose; FBI, fasting blood insulin; HbA1c, glycated hemoglobin; HDL-C, high-density lipoprotein cholesterol; HOMA-IR, homeostasis model assessment of insulin resistance; LDL-C, low-density lipoprotein cholesterol; RCT, randomized controlled trial; SBP, systolic blood pressure; TC, total cholesterol; TG, total triglycerides; T Bili, total bilirubin; WC, waist circumference.

**Table 3:** PICOS criteria for inclusion and exclusion of studies.

Parameter	Defined Criteria for the Current Study
P (population)	Adult patients with MAFLD
I (intervention)	Curcumin supplementation or curcumin supplementation with lifestyle advice (e.g., diet and/or physical activity and/or other lifestyle modifications)
C (comparison)	No special comparison criteria
O (outcomes)	Changes in biochemical parameters or anthropometric measurements
S (study design)	Randomized controlled trial

**Table 5:** Pooled effect sizes based on curcumin supplementation and curcumin supplementation with other advice in treating MAFLD.

Outcomes	MD	95% CI	*p*-Value	I^2^
ALT (U/L)	−5.3047	−8.7520, −1.8573	0.0026	6.0
AST (U/L)	−3.5597	−5.7556, −1.3638	0.0015	17.5
FBI (µU/mL)	−1.1430	−1.5439, −0.7421	<0.0001	9.3
HOMA-IR	−0.2884	−0.3950, −0.1817	<0.0001	0
TG (mg/dL)	−12.6001	−22.9311, −2.2692	0.0168	0
TC (mg/dL)	−15.7896	−28.2686, −3.3106	0.0131	64.6
LDL-C (mg/dL)	−14.1699	−25.9117, −2.4281	0.0180	70.8
WC (cm)	−2.6303	−4.9350, −0.3256	0.0253	0

## Data Availability

Not applicable.

## Supplementary Materials

The following supporting information can be downloaded at: https://www.mdpi.com/article/10.3390/ijerph20054266/s1. Figure S1: Changes in ALT level. A funnel plot showing the effect estimates from 12 individual studies against their standard errors regarded as some measure of each study’s size or precision; Figure S2: Changes in AST level. A funnel plot showing the effect estimates from 11 individual studies against their standard errors regarded as some measure of each study’s size or precision.
