# Supplementary material for: Meta-Analysis of Exploring the Effect of Curcumin Supplementation with or without Other Advice on Biochemical and Anthropometric Parameters in Patients with Metabolic-Associated Fatty Liver Disease (MAFLD)
Source: Int J Environ Res Public Health. 2023 Feb 27;20(5):4266. doi: 10.3390/ijerph20054266 (PMC10001478; doi:10.3390/ijerph20054266)
Supplement: Supplementary file 1 [file ijerph-20-04266-s001.zip › ijerph-2228298-supplementary.pdf]

International Journal of
Environmental Research and Public Health

MDPI

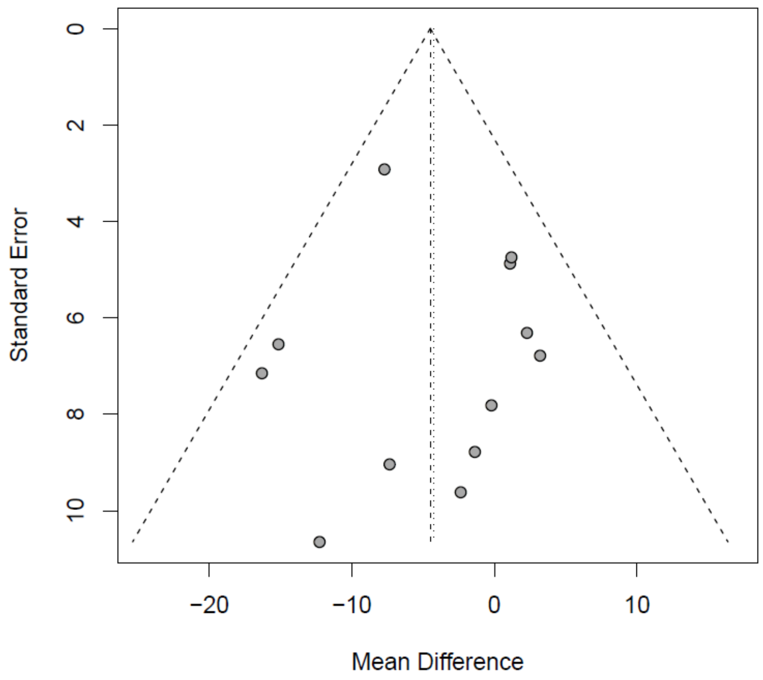

Figure S1. Changes in ALT level. A funnel plot showing the effect estimates from 12 individual studies against their standard errors regarded as some measure of each study's size or precision.

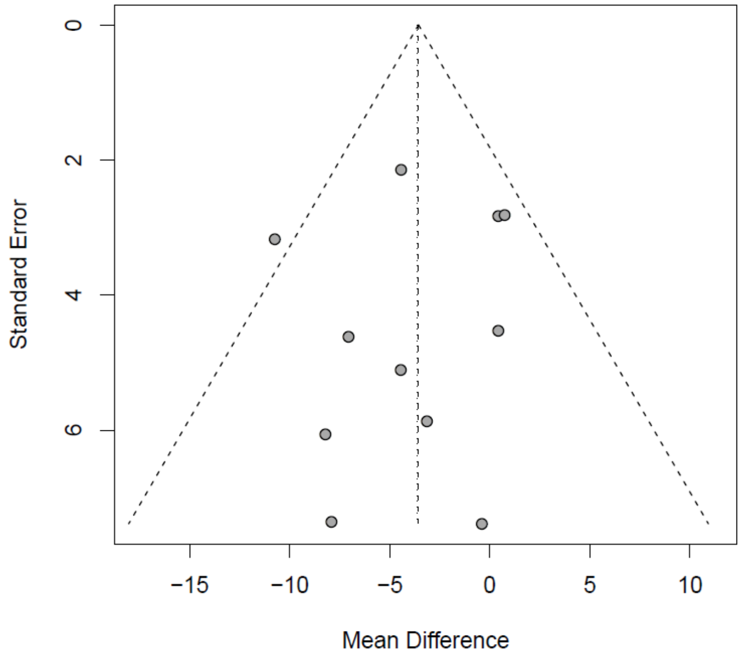

Figure S2. Changes in AST level. A funnel plot showing the effect estimates from 11 individual studies against their standard errors regarded as some measure of each study's size or precision.

Figures S1 and S2 represent funnel plots which are widely used graphical tools for discovering publication bias. If the bias and between study heterogeneity are absent then the plot should look like a symmetrical inverted funnel. Figure S1 includes 12 studies on ALT and Figure S2 – 11 studies on AST. Some of the studies had to be excluded due to insufficient data.
